# Plasmodium Parasitaemia and Urine Alterations among Pregnant Women Attending Antenatal Care in Aba Metropolis, Abia State, Nigeria

**DOI:** 10.1155/2024/6681943

**Published:** 2024-04-04

**Authors:** Paula Melariri, Chioma Ihemanma, Udoka Chigbo, Kirstie Eastwood, Chika Uche, Paschal Etusim

**Affiliations:** ^1^Department of Environmental Health, School of Behavioural and Lifestyle Sciences, Faculty of Health Sciences, Nelson Mandela University, Gqeberha 6019, South Africa; ^2^Unit of Medical Parasitology and Entomology, Department of Biology/Microbiology, Abia State Polytechnic, Aba, Abia State, Nigeria; ^3^Unit of Medical Parasitology and Entomology, Department of Animal and Environmental Biology, Faculty of Biological Sciences, Abia State University, Uturu, Nigeria; ^4^Department of Medical Microbiology, College of Medicine, Imo State University, Owerri, Nigeria; ^5^Unit for Statistical Consultation, Research Development, Nelson Mandela University, Gqeberha 6019, South Africa; ^6^Department of Haematology, College of Medicine, Abia State University, Uturu, Nigeria

## Abstract

Malaria presents a huge threat to pregnant women, their foetus, and children below five years. This study is aimed at assessing malaria prevalence, associated clinical symptoms, and urine abnormalities among pregnant women in Aba metropolis, Abia State, Nigeria. A cross-sectional study involving 450 pregnant women purposively selected from nine health care centres was conducted. Data were analysed using SPSS version 26. The overall malaria prevalence rate was 68.4% (*n* = 308). Age group of 21-25 years had the highest prevalence rate of 20.4% (*n* = 92) while the least was recorded among the age group of 41-45 years (17 (3.8%)). Pregnant women in their first trimester had the greatest prevalence rate of 28.6% (*n* = 129), and the least prevalence was recorded among those in their third trimester (7 (15.6%)). The primigravidae were mostly infected at the rate of 27.7% (*n* = 125) whereas the multigravidae recorded the least prevalence of 85 (18.9%). Participants with secondary education were the most susceptible at the rate of 38.6% (*n* = 174). The infected participants exhibited significantly higher frequencies of reported fever (*p* > 0.001, OR 12.881, 95% CI 3.977-41.725) and headaches (*p* < 0.001, OR 4.688, 95% CI 1.819-12.083). However, cold, cough, body pains, poor appetite, and catarrh showed no significant association with malaria infection at *p* > 0.05. Participants using long-lasting insecticidal nets (LLINs) showed significantly lower prevalence rate of malaria infection (*p* < 0.001, OR 2.485, 95% CI 1.619-3.814). Malaria-infected participants showed statistically significant frequencies of proteinuria (*p* < 0.001, OR 274.14, 95% CI 16.91-4444.0), bilirubinuria (*p* < 0.001, OR 49.29, 95% CI 11.01-186.34), and urobilinogenuria (*p* < 0.001, OR 65.16, 95% CI 4.00-1062.40) than those not infected. Ascorbic acid, nitrate, and ketone showed no significant associations with malaria infection at *p* > 0.005. Infected participants had statistically significant amber and clear urine colour whereas there was no statistically significant difference between the pH levels of urine of the malaria-infected and malaria-noninfected pregnant women.

## 1. Introduction

Despite the efforts to curb the menace of malaria infection in young children and pregnant women, more persons are getting infected, and according to WHO malaria report (2023), there were estimated 249 million cases globally, exceeding the prepandemic level of 233 million in 2019 by 16 million cases. Significant increases were also observed in Nigeria and other African countries like Ethiopia, Papua New Guinea, and Uganda [1]. About 94,070 deaths amounting to 23% of deaths globally have been recorded in pregnant women and children under five years of age [2]. In 2021, Nigeria accounted for 38.4% of malaria deaths globally in children below 5 years, a significant increase from 2019 [3].

Abia State as in most parts of Nigeria is prone to clustering cases of malaria mostly driven by climatic, ecological, and human factors [4] especially in localities with limited health facilities. There is need for surveillance and critical interventions in these localities with asymptomatic cases of malaria to ensure effective diagnostics and therapeutics for effective control of malaria parasitaemia.

This research is aimed at studying malaria prevalence, urine abnormalities, and clinical symptoms of pregnant women in Aba metropolis. The specific objectives included the following:

1. To determine malaria parasite prevalence among the pregnant women
2. To investigate urine abnormalities and clinical symptoms among the infected participants
3. To evaluate malaria control measures among the pregnant women in Aba metropolis

Urine parameters are a set of properties that are detected in urinalysis. Urinalysis provides semiquantitative determinants of glucose, bilirubin, ketone, blood, protein, urobilinogen, pH, and nitrite, and it gives useful information and insights on the state of carbohydrate metabolism, kidney function, liver function, acid-base concentration, and urinary tract infection. Abnormal urinary observations like proteinuria, bilirubinuria, ketonuria, urobilinogenuria, and haematuria point to the body's inner workings and reflect different diseases. Some studies have observed proteinuria, bilirubinuria, and urobilinogenuria with malaria infection [5–7]. Data and inferences from the study can be used to guide health policies associated with malaria diagnostics, therapeutics, and control measures in Abia State.

This paper presents findings on laboratory investigations of malaria prevalence and urine abnormalities among pregnant women that attended the selected ANCs in Aba metropolis.

## 2. Methodology

This study was conducted in nine health care facilities in Aba which included PHC I (Osisioma Ngwa LGA), PHC II (Aba North LGA), PHC III (Aba North LGA), PHC IV (Osisioma Ngwa LGA), PHC V (Aba North LGA), PHC VI (Aba South LGA), PHC VII (Aba South LGA), PHC VIII (Aba South LGA), and PHC IX (Aba South LGA). The climate is tropical with a dry season and rainy season. Malaria transmission here is variable with peak intensity in the rainy season with consequent morbidity. This structured clinical study was conducted from February to August 2019. Abia State covers a space of 6,320 km^2^ and approximately resident population of 3 million [8]. It is located between longitude 07° 00 and 08° 10 east and latitude 04° 45° and 06° 07° with three senatorial districts and 17 LGAs. Aba is subdivided into two local government areas known as Aba South and North and is known as the commercial centre in southeast Nigeria [9]. Mean annual temperature ranges from 22.5 to 31.9°C. The main vector for transmission of malaria is *Anopheles gambiae* which breeds favourably due to the high humid conditions and characteristic stagnant water pools seen all around during the rainy season and unhygienic waste disposal system. Malaria transmission is perennial and is hyperendemic in most localities of Abia State.

### 2.1. Study Design

This cross-sectional descriptive study was conducted in nine primary health care centres (50 pregnant women from each of the five primary health care centres) that offer antenatal services to pregnant women. Eligibility criteria included pregnant women of different gravidity and gestation stages who attended their antenatal visits within the ages of 16 years to 45 years, who were not on antimalarial drug as at the time of the study, those who were HIV negative and without sickle cell anaemia, and those without renal or liver disorders. Enrollment of cohorts for this study was done within the study area from February to August 2019. Urinalysis and malaria detection were conducted on the 450 women using the Giemsa-stained thick blood film microscopy. Two trained microscopists in the microbiology unit of ABSUTH examined the slides independently under oil immersion (100x) objective with discrepancies resolved by a third reader. Alterations in urine samples observed during testing were compared with clinical signs in symptomatic and asymptomatic study participants.

### 2.2. Ethical Consideration

Ethical clearance for the study was obtained from Abia State Ministry of Health, Umuahia (ethical clearance certificate no. AB/MH/AD/904/T), and Abia State Primary Health Care Development Agency (ABIA SPHCDA), Umuahia (ethical clearance certificate number AB/PHCDA/157/XXX). These clearance letters which detailed the objectives of this study enabled access to pregnant women in the local government councils and primary health centres mapped out for the study. Informed consent was sought from eligible subjects who were enrolled after their due consent was received. Staff of these antenatal clinics assisted in sample collection and administering pretested structured questionnaires which included information on sociodemographics, malaria control measures used at home, and possible clinical malaria symptoms.

### 2.3. Sample Size Determination

**Figure 1:**
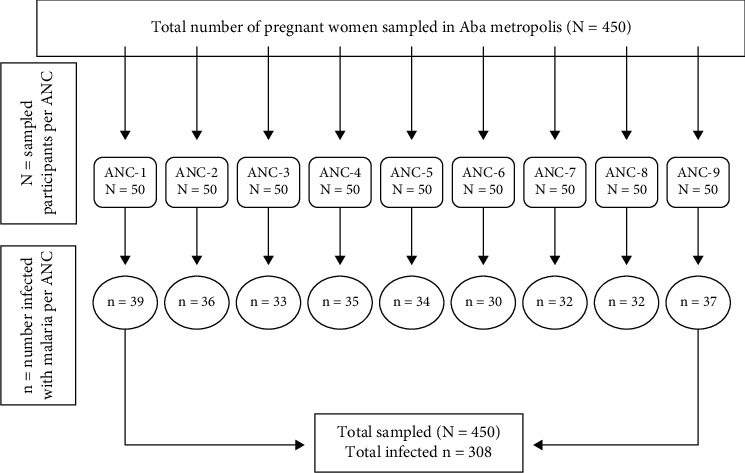
Flow chart indicating the sampling procedure.

A suitable sample size (*N* = 450) (Figure 1) of the pregnant women in Aba metropolis was derived as follows.

A prevalence rate of 40% [10] was chosen. Margin of a sampling error or precision tolerated was set at 5%, at 95% confidence interval using the formula

(1) $$\begin{array}{c} n=\frac{Z^{2}P\left(1-P\right)}{d2}, \end{array}$$

where *n* is the sample size, *Z* statistic = 1.96 (statistical constant), *p* = 40% (prevalence: population based), and *d* = 0.05% (marginal error or precision) [11].

### 2.4. Sample Collection

Blood sampling via venipuncture was employed by registered nurses in the nine antenatal clinics used for the study to collect 2 mL of venous blood from the arm of each subject using a sterile syringe. Collected blood samples were transferred into sterile EDTA (ethylene diamine tetra acetic acid) and transported to the Parasitology Laboratory of the Abia State University Teaching Hospital (ABSUTH) for analysis. Also, 10 mL of clean-catch urine samples was collected in sterile universal bottle from each study participants, labelled, and assayed with Medi-Test Combi 9® urine test strip, within one hour of collection [5, 6].

### 2.5. Blood Smear Microscopy

Thick blood smears were prepared and interpreted using modified method described by WHO guideline for preparation, staining and reading of malaria blood slides [12]. 6 *μ*L of blood was spread in a diameter of 15 mm of a grease-free slide and air-dried. The blood smears were stained with 10% Giemsa stain. Stained smears were air-dried and examined using objective eye piece (10x and 100x oil immersion lenses) of Olympus binocular microscope (Olympus Tokyo, Japan) to detect the presence of malaria parasites. A case was defined as a pregnant woman aged 16-45 years coming for ANC at any of the preselected PHCs with or without malaria symptoms who tested positive for malaria.

### 2.6. Data Analysis

In this study, continuous variables were expressed as percentages and means, and evaluated using Student's *t*-test. Pearson's chi-square or Fisher's exact test was used to compare categorical variables while chi-square test was used to determine associations between demographic profile of study participants. Odds ratio (OR) was used to compare the relative odds of malaria infection to control measures. All analyses were performed using SPSS version 26 (IBM Corp., New York, USA). The level of significance was set at *p* < 0.05.

## 3. Results

Table 1 shows the sociodemographic characteristics and malaria prevalence among the study participants. A total of 450 pregnant women participated in the study. The overall prevalence of malaria among the pregnant women in the study area was 308 (68.4%). Nine centres were studied; PHC I, in Osisioma Ngwa LGA, had the highest prevalence rate of 39 (8.7%) while PHC VI, also in Osisioma Ngwa LGA, had the lowest of 30 (6.7%). The age group of 21-25 years showed the highest malaria prevalence of 92 (20.4%) while the least was recorded in the age group of 41-45 years (17 (3.8%)). Women in their first trimester had the highest malaria prevalence rate of 129 (28.6%) whereas the least prevalence rate was recorded among pregnant women in their third trimester (70 (15.6%)). As shown in the table, the primigravidae showed the highest malaria prevalence rate of 125 (27.7%) while the least was observed among the multigravidae (85 (18.9%)). The subjects with secondary education had the highest malaria prevalence rate of 174 (38.6%) while the least was observed among those with no formal education (15 (3.3%)). A higher malaria prevalence was recorded among married pregnant women (288 (64.0%)) than single women (20 (4.4%)).

167 of the women sampled showed varying clinical symptoms while 283 were asymptomatic. Of these, 158 (35.1%) of the symptomatic participants were malaria infected while 150 (33.3%) of the asymptomatic participants were infected with malaria. The common symptoms at presentation included fever, cold, cough, headache, body pains, poor appetite, and catarrh (Table 2). A bivariate analysis using chi-square tests of association was conducted between malaria infection and the reported symptoms. The participants infected with malaria exhibited significantly higher frequencies of reported fever (*p* < 0.01, OR 12.881, 95% CI 3.977-41.725) and headache (*p* < 0.01, OR 4.688, 95%CI 1.819-12.083). Cold, cough, body pains, poor appetite, and catarrh showed no significant associations with malaria infection (*p* > 0.05).

A bivariate analysis using chi-square tests of association was conducted between malaria infection and the presence of urine markers (Table 3). The participants infected with malaria exhibited significantly higher frequencies of proteinuria (*p* < 0.001, OR 274.14, 95% CI 16.91-4444.00), bilirubinuria (*p* < 0.001, OR 45.29, 95% CI 11.01-186.34), and urobilinogenuria (*p* < 0.001, OR 65.16, 95% CI 4.00-1062.40) than those not infected. Ascorbic acid, blood, nitrate, and ketone showed no significant associations with malaria infection (*p* > 0.05). Bivariate analysis of malaria preventive measures in this study revealed significant positive associations with control measures (OR 81.818) and use of LLINs (OR 2.485). Positive significance was observed between MiP infection and use of LLINs (*p* < 0.001) and control measures (*p* < 0.001). A positive relationship was observed between urine macroscopy (*p* < 0.001) and MiP (*p* < 0.05) (Table 4). The urine colours of most infected subjects (236/450) were pale amber and clear (PAC) (Table 5). Three hundred and eight subjects that were positive for malaria had urine pH 6.49 which was not significant to malaria infection at *p* < 0.001.

## 4. Discussion

The present study is aimed at identifying abnormal urine factors associated with *falciparum* malaria among parturient women aged 16-45 years attending ANCs in nine PHCs located in 3 LGAs that make up Aba metropolis with variable malaria transmission confirmed with microscopy and RDTs. Malaria parasitaemia in the studied population was found to be significantly high at 68.7%. Pregnant women in this study area are constantly exposed to infected mosquito bites due to the favourable climatic conditions and mosquito breeding sites in the area. Houses in this area are characterized by use of several water storing containers to cope with water scarcity. These containers can form mosquito breeding sites in homes. More so, living houses are sited around bushes and farmlands with plants ecologically suitable for mosquito breeding, such as cocoyam and plantain. These factors and more encourage the multiplication of malaria vectors. The finding compares with the malaria prevalence rates found by other researchers in Ebonyi State, Port Harcourt, Umuahia, Southern Ethiopia, Uganda, and Sudan, respectively [13–17]. Four major factors have been observed by previous studies that determine the spread of malaria in pregnancy, and they include environmental, vectoral, parasite, and host factors [13]. In 2017, a malaria prevalence of 40.1% was observed in pregnant women in the same study area [10]; however, according to the most recent malaria report of WHO [1], there is up to 16 million malaria cases postpandemic increase globally, most of which were recorded in African countries like Nigeria, Ethiopia, Papua New Guinea, and Uganda. Furthermore, another recent study conducted in Imo State (a neighbouring state with similar ecological, environmental, and climatic factors) observed a very similar malaria prevalence of 63.7% in 2023 among pregnant women [18].

Age groups of 21-25 years had the highest prevalence rate of 92 (20.4%). This compares well with the findings of [13] and also [14] who reported the age group of 21-25 years to be the most susceptible to malaria. Immune responses have been found to increase by recurrent malaria infection as individuals get older [17]. Studies have reported that semi-immunity to malaria infection develops based on the cumulative exposure to the parasite [19]. However, this contradicts the findings of [14], who observed higher prevalence rates among older pregnant women of age groups 28-32 and 43-47 years. The highest malaria prevalence rate of 129 (28.6%) was observed among the pregnant women in their 1^st^ trimester and lowest among the pregnant women in their third trimester (70 (15.6%)). This finding is in line with the findings of other researchers [5, 6], who also observed highest malaria prevalence rates among pregnant women in their first trimester. Furthermore, women in the third trimester may have received some doses of sulfadoxine-pyrimethamine intermittent preventive treatment of malaria in pregnancy. The pregnant women possibly had lowered immunity from the sudden onset of pregnancy, the baseline immunity acquired in endemic areas notwithstanding. With respect to parity, malaria prevalence was highest among the primigravidae (125 (27.7%)). This could be due to the development of new uteroplacental vasculature during the 1^st^ pregnancy which has no pre-exposure to malaria infection and therefore immunologically naïve and susceptible [20]. More so, early onset of efficient antibody response in multigravidae and the delayed production of antibodies in primigravidae may account for the gravidity dependent and differences in prevalence rates of malaria infection among pregnant women [14]. This finding is in agreement with the findings of other researchers [5, 6]. 30 women out of the 250 examined were single mothers of which 20 (4.4%) were infected with malaria while 288 (64.0%) out of 420 married women were infected.

In this study, the highest prevalence of malaria with respect to educational status was observed among those with secondary education (174 (38.6%)), followed by the pregnant women with tertiary education (84 (18.7%)), whereas women with primary education recorded 35 (7.8%) and the least prevalence rate was found in those with no formal education (15 (3.3%)), which could be attributable to the small number of this group. The lower infection rate observed among pregnant women with tertiary education could be attributed to their higher living standards, awareness, and better use of preventive measures against malaria infection. This study also revealed that the use of malaria preventive measures conferred some levels of protection to the users but did not eliminate the infection entirely (Table 5), as reported in the findings other researchers like [5, 6, 21]. Thus, the correct use of integrated control measures is recommended. The malaria prevalence rate (150 (33.3%)) seen among 283 asymptomatic women is quite disturbing. Several studies have also shown prevalence of asymptomatic malaria among pregnant women and association with anaemia, still birth, poor pregnancy outcome, and other consequences of malaria infection in pregnancy [22]. Following the observations of [23–25], clinical manifestation of malaria in pregnancy varies between individuals. Signs and symptoms may span from mild fever in uncomplicated malaria to life-threatening conditions with severe anaemia acute respiratory distress syndrome, hypoglycaemia shock, metabolic acidosis, acute kidney injury, and cerebral malaria in individual with severe malaria. Fever and headache as symptoms are widely observed and used to predict malaria episode as an established proxy of malaria transmission due to its association with increase of body temperature. This study documented significantly higher urinary protein, bilirubin, and urobilinogen in malaria-infected pregnant women, indicating a high suspicion for malaria infection in pregnancy, even in the face of negative blood film. Haemolysis of malaria parasitized and nonparasitized red blood cells is considered as an important factor causing mild jaundice, hence the appearance of bilirubin in urine.

The evaluation of urinalysis in malaria infections among pregnant women enhances knowledge of host factors that enable detection of MiP. In this study, 100% and 98.37% of infected subjects were positive for bilirubinuria and proteinuria, respectively, which correlates with previous reports [5–7, 9]. A lower prevalence of 24.3% was reported by [7]. A study of anaemic pregnant women attending ANC in Ghana reported a significantly lower level of bilirubin. Low bilirubin among malaria-infected pregnant women is mostly due to the haemolysis of the red cells which is associated with changes in haematological and urine characteristics. Variations in proteinuria levels among subjects in this study and previous studies may be associated with differences in locations, sample populations, age of cohorts, demography, study duration, lifestyle, duration of study, and health status of subjects. Proteinuria in malaria patients has been associated with bilirubinuria, urobilinogenuria, and blood in urine which agrees with the findings of this work [5, 6, 26, 27].

This epidemiological study on urine abnormalities in MiP may provide better understanding of common changes in urine factors and its related outcomes in infected pregnant women. Among urine factors associated with *P. falciparum* infections among pregnant women, proteinuria was the most prevalent among subjects along with other key indicators examined.

The pathophysiology of severe malaria with manifestation of acute kidney injury is an ongoing study. In this study, urine markers were found to be associated with acute malaria infection. Infected women with ascorbic acid blood and ketone in their urine were not significant in relation to malaria infection. Urine abnormalities associated with kidney disorder have been observed with *falciparum* malaria [28] which usually result in haemodynamic dysfunction and immune response. Also, proteinuria and bilirubinuria associated with kidney involvement in malaria [29] may be responsible for malaria-specific proteins to be detected in the urine of pregnant women in this study. If these proteins could be excreted in urine due to their molecular weight, then malaria antigens like *pfHRP*2 could be freely excreted as well [28]. Anaemia caused by *P. falciparum* results in the destruction of red cells containing these obligatory intraerythrocytic parasite and also uninfected red cells [17]. Urinalysis, though not an alternative diagnostic tool for malaria infection, may aid clinicians working in malaria endemic resource-limited countries as the first line care for timely recognition of patients at risk, allowing prompt care to reduce the dangerous consequences of malaria in pregnancy. Urinary abnormalities like proteinuria, bilirubinuria, urobilinogenuria may assist in identifying patients with severe malaria infection as it detects pathological changes in malaria patients. However, the limitations in terms of the number of pregnant women accessible in the various health care centres could be attributed to the inability to access the health care centres due to lack of access roads and financial constraints. However, the nine centres which participated in the study are relatively busy centres as they enjoy better patronage. Another limitation is the possibility that certain physiological conditions could stimulate similar urinary parameters as seen in the malaria positive pregnant women. More so, the study could not include observations in urine alterations in malaria-infected nonpregnant women to determine if the changes were exacerbated by pregnancy.

## 5. Conclusion

Malaria prevalence in Aba metropolis is significantly high among pregnant women. Thick blood film microscopy remains the standard diagnostic method for malaria diagnosis. Integrated protective measures conferred appreciable level of protection on the pregnant women against malaria. Urine abnormalities like bilirubin, proteinuria, and urobilinogenuria in pregnancy should be further investigated for malaria infection even in asymptomatic situation to avoid the adverse effects of malaria in pregnancy.

## Figures and Tables

**Table 1:** Sociodemographic characteristics and malaria prevalence among study participants.

Characteristics	Not infected		Infected		Total	
	Number	%	Number	%	Number	%
Centre						
PHC I (Osisioma Ngwa LGA)	11	2.4	39	8.7	50	11.1
PHC II (Aba North LGA)	14	3.1	36	8.0	50	11.1
PHC III (Aba North LGA)	17	3.7	33	7.3	50	11.1
PHC IV (Osisioma Ngwa LGA)	15	3.3	35	7.8	50	11.1
PHC V (Aba North LGA)	16	3.6	34	7.6	50	11.1
PHC VI (Osisioma Ngwa LGA)	20	4.4	30	6.7	50	11.1
PHC VII (Aba South LGA)	18	4.4	32	7.1	50	11.1
PHC VIII (Aba South LGA)	18	4.0	32	7.1	50	11.1
PHC IX (Aba South LGA)	13	2.9	37	8.2	50	11.1
Total						
Aba	142	31.4	308	68.4	450	100
Age group						
16-20 years	4	0.8	20	4.4	24	5.3
21-25 years	25	5.5	92	20.4	117	26.0
26-30 years	50	11.1	83	18.4	133	29.5
31-35 years	30	6.7	62	13.8	92	20.4
36-40 years	28	6.2	34	7.6	62	13.7
41-45 years	5	1.1	17	3.8	22	4.8
Gestation						
1st trimester	44	9.8	129	28.6	173	38.4
2nd trimester	46	10.2	109	24.2	155	34.4
3rd trimester	52	11.5	70	15.6	122	27.1
Parity						
Primigravidae	50	11.1	125	27.7	175	38.8
Secundigravidae	58	12.9	98	21.8	156	34.7
Multigravidae	34	7.5	85	18.9	119	26.4
Educational status						
No formal education	10	2.2	15	3.3	25	5.6
Primary education	25	5.6	35	7.8	60	13.3
Secondary education	56	12.4	174	38.6	230	51.1
Tertiary education	51	11.3	84	18.7	135	30.0
Marital status						
Single	10	2.2	20	4.4	30	6.7
Married	132	29.3	288	64.0	420	93.3

**Table 2:** Associations between malaria infection and reported clinical symptoms.

Symptoms	Not infected		Infected		Chi-square test					95% CI	
	Number	%	Number	%	Value	Df	*p* value	Cramer's *V*	OR	Lower	Upper
Fever											
No	139	36.60	241	63.40	28.542	1	<0.001	0.252	12.881	3.977	41.725
Yes	3	4.30	67	95.70							
Cold											
No	139	32.40	290	67.60	3.042	1	0.081	N/A	2.876	0.833	9.927
Yes	3	14.30	18	85.70							
Headache											
No	137	34.30	263	65.80	12.01	1	0.001	0.164	4.688	1.819	12.083
Yes	5	10.00	45	90.00							
Cough											
No	137	31.90	293	68.10	0.416	1	0.519	N/A	1.403	0.500	3.938
Yes	5	25.00	15	75.00							
Body pains											
No	141	32.30	296	67.70	3.53	1	0.060	N/A	5.716	0.736	44.397
Yes	1	7.70	12	92.30							
Poor appetite											
No	139	32.40	290	67.60	3.042	1	0.081	N/A	2.876	0.833	9.927
Yes	3	14.30	18	85.70							
Catarrh											
No	139	32.40	290	67.60	30.42	1	0.081	N/A	2.876	0.833	9.927
Yes	3	14.30	18	85.70							

**Table 3:** Associations between malaria infection and presence of various urine markers.

	Not infected		Infected		Chi-square test					95% CI	
	No.	%	No.	%	Value	Df	*p* value	Cramer's *V*	OR	Lower	Upper
Proteinuria											
Negative	143	47.49	157	52.51	104.775	1	<0.001	0.483	274.14	16.91	4444.00
Positive	0	0.00	151	100.00							
Bilirubinuria											
Negative	140	42.81	187	57.19	70.203	1	<0.001	0.395	45.29	11.01	186.34
Positive	2	1.63	121	98.37							
Ascorbic acid											
Negative	110	30.64	249	69.36	0.688	1	0.407	N/A	0.82	0.51	1.33
Positive	32	35.16	60	64.84							
Urobilinogenuria											
Negative	142	36.13	251	63.87	30.091	1	<0.001	0.259	65.16	4.00	1062.40
Positive	0	0.00	57	100.00							
Blood											
Negative	141	32.41	294	67.59	4.451	1	0.035	0.099	6.71	0.87	51.57
Positive	1	6.67	14	93.33							
Nitrate											
Negative	140	31.39	306	68.61	0.636	1	0.425	N/A	0.46	0.06	3.28
Positive	2	50.00	2	50.00							
Ketone											
Negative	140	31.46	305	68.54	0.636	1	0.425	N/A	0.69	0.11	4.17
Positive	2	40.00	3	60.00							

**Table 4:** Associations between malaria infection and urine macroscopy/appearance.

	Not infected		Infected		Chi-square test			
	No.	%	No.	%	*p* value	Df	*p* value	Cramer's *V*
Urine								
AC	3	4.60	62	95.40	33.799	3	<0.001	0.274
PAC	136	36.60	236	63.40				
PACL	3	75.00	1	25.00				
DAC	0	0.00	9	100.00				

Keys: AC: amber and clear; PAC: pale amber and clear; PACL: pale amber and cloudy; DAC: dark amber and clear.

**Table 5:** Malaria prevalence with respect to various malaria preventive measures used.

	Not infected		Infected		Chi-square test					95% CI	
	No.	%	No.	%	Value	Df	*p* value	Cramer's *V*	OR	Lower	Upper
Use LLINS											
Yes	102	39.5	156	60.5	17.826	1	<0.001	0.199	2.485	1.619	3.814
No	40	20.8	152	79.2							
Control measures											
None	5	19.20	21	80.80	81.818	6	<0.001	0.426			
Close windows	2	18.20	9	81.80				
Window nets	4	8.70	42	91.30				
LLINs	25	20.00	100	80.00				
Insecticides	5	8.80	52	91.20				
LLINs and others	75	56.80	57	43.20				
Multiple	26	49.10	27	50.90				

Key: LLINs: long-lasting insecticidal nets.

## Data Availability

The data was not stored in a repository but could be available electronically upon reasonable request from the corresponding author.
